# Safety and Tolerability of Ketamine Use in Treatment-Resistant Bipolar Depression Patients with Regard to Central Nervous System Symptomatology: Literature Review and Analysis

**DOI:** 10.3390/medicina56020067

**Published:** 2020-02-09

**Authors:** Adam Włodarczyk, Wiesław Jerzy Cubała

**Affiliations:** Department of Psychiatry, Faculty of Medicine, Medical University of Gdańsk, 80-952 Gdańsk, Poland; cubala@gumed.edu.pl

**Keywords:** ketamine, TRBD, BD, bipolar depression, central nervous system, safety, dissociation, CADSS, BPRS

## Abstract

The current psychopharmacological treatment approaches for major depression focus on monoaminergic interventions, which are ineffective in a large proportion of patients. Globally, treatment-resistant bipolar depression (TRBD) affects up to 33% of depressive patients receiving treatment. Certain needs are still unmet and require new approaches. Many studies are in favor of treatments with ketamine, an N-methyl-D-aspartate (NMDA) receptor antagonist, even in single use, whose effects emerge in minutes to hours post administration. However, little data are available on ketamine performance in TRBD patients with somatic comorbidities, including highly prevalent ones, i.e., cardiovascular disease (heart failure, hypertension, post-myocardial infarct, arrhythmias, etc.) diabetes, and obesity, and depression-associated comorbidities such as stroke, epilepsy, as well as in the elderly population. The literature shows that treatment with ketamine is efficacious and safe, and the majority of adverse drug reactions are mild and tend to mostly disappear within 30 min to 2 h of ketamine administration.

## 1. Introduction

Bipolar disorder is considered a major health problem worldwide because of the increased rates of premature mortality and disability [1]. Unfortunately, the broad choice of mood stabilizers and antipsychotic drugs, often prescribed to treat bipolar depression with several pharmacodynamic profiles, along with non-pharmacological interventions including electroconvulsive therapy and psychotherapy, still cannot fully address the burden of the disease. The current psychopharmacological treatment approaches in depression mainly focus on mood stabilizers, second-generation antipsychotics, and monoaminergic interventions, but a significant proportion of patient do not respond to them adequately. Treatment-resistant bipolar depression (TRBD) patients who do not achieve remission are up to 33% of the depressive patients global count [2,3,4]. Delayed onset of the clinical action of the antidepressants, tolerability of the drugs, comorbidities, and low drug efficacy in some patients are the key unmet challenges of contemporary psychiatry [5]. Nonetheless, recent ketamine and esketamine research has resulted in promising outcomes in TRBD treatment [3,6].

The aim of this paper is to review current concepts and data on the treatment of drug-resistant symptoms.

## 2. Ketamine in Major Depressive Disorder (MDD) and Bipolar Depressive Disorder

Ketamine, an N-methyl-D-aspartate (NMDA) receptor antagonist, is known since 1963 when it was introduced in anesthesia and is characterized by safety and rapid action [7]. It has recently been investigated as an antidepressant in treatment-resistant depression (TRD) patients and received Food and Drug Administration (FDA) approval in March 2019. Along with its introduction in the US market, safety concerns arose regarding the use of this medication, particularly in patient populations characterized by comorbidities, concomitant medication, and possible adverse events of the administered therapies.

Ketamine, as both a racemic mixture of R- and S-ketamine in a 1:1 ratio and the S-enantiomer, acts as an N-methyl-D-aspartate receptor (NMDA) receptor antagonist [3]. There is a limited number of case reports indicating that esketamine exerts similar antidepressant effect as ketamine, with a better tolerability profile [8]. Its intravenous formulation is more popular, being widely used in anesthesia, but oral and subcutaneous administrations are more convenient [9]. Drug dosing differs depending on the administration route and pattern. The inhaled/intranasal form has shown efficacy at the dose range of 28 to 84 mg administered twice weekly, with a significantly ascending dose–response relationship [6,10], while the intravenous formulation is usually administered at 0.5 mg/kg over 40 min [11,12,13]. There are studies showing efficacy of the drug at lower doses (0.1–0.25 mg/kg over 40 min infusion) [9,14] as well as at higher doses in the range of 0.7 mg/kg [9]. Interestingly, in a study of 14 TRD patients given low ketamine doses in the sublingual form (10 mg s.l.), no efficacy for the treatment of TRBD was seen, and adverse drug reactions were observed [15]. Intra-muscular ketamine doses of 50 mg given every 3, 4 days showed response after 7 days, but on a follow-up in the eight week and later, there was no antidepressant effect in TRBD patients [3]. No response was also noted when an intramuscular form of 100 mg was administered TRBD patients [9]. This could be due to different plasma levels of the drug, which depend on the route of administration [16].

We searched the PubMed, Scopus, Medline, Science Citation Index, and Google Scholar databases for articles in English language without limits on publication year containing the following keywords: “ketamine”, “dissociation”, “depression”, “dissociative”, “adverse events”. To explore the central nervous system adverse events related to ketamine described in the selected articles, no specific keywords were used, as the selected literature was further explored by the authors independently, using other keywords. 

Several drug administration time patterns were employed in the examined studies, with the majority using 40 min i.v. infusions. Rapid and extensive administrations regimens were described, ranging from 2 to 5 min (i.v.) [16] and up to 100 min, respectively [9]. The FDA and the European Medicines Agency (EMA) established that esketamine intake via intranasal administration takes ca. 15 min (one spray of esketamine self-administered into each nostril in 1, 2, or 3 points, corresponding to 28, 56, or 84 mg, respectively, each separated by 5 min) [6,17].

The intravenous administration of ketamine is the best studied administration route, but market opportunities exist also for forms of ketamine administered orally. These, in fact, have lesser bioavailability (ca. 20–25% of the drug reaches the bloodstream) but are easier to administer [18] and are more affordable than intranasal esketamine. Three randomized controlled trials, with doses from 0.25 up to 7 mg/kg of body mass weight, showed effectiveness of oral ketamine in treating TRD patients with severe depression with or without suicidal ideation [18]. However, in one study the results were rather modest, because 30% of the patients receiving oral ketamine showed ‘some benefit’, whereas the remaining 70% did not experience any change or even presented worsening of mood [19]. There is other evidence showing that the effects of oral ketamine are not as rapid as those associated with intravenous administration [20]. Although these results are promising, there are still no data regarding the effective dose, as the current dosing varies from 2.0 up to 2.5 mg/kg of body mass weight. More studies examining the effects of different doses are needed.

### 2.1. Special Populations

Little data are available on ketamine performance in TRBD patients with somatic comorbidities, who receive concomitant medication or present with a history of major medical events. Cardiovascular disease (heart failure, hypertension, post-myocardial infarct, arrhythmias, etc.), diabetes, obesity, depression-associated comorbidities, such as stroke and epilepsy, as well as old age are often present in TRD patients [21,22].

### 2.2. Central Nervous System Safety of Ketamine in Depression Treatment

To evaluate central nervous system safety, most researchers use the same scales, such as the Clinician Administered Dissociative States Scale (CADSS) and the Brief Psychiatric Rating Scale (BPRS) [11,12,14,15]; the Physician Withdrawal Checklist (PWC-20) is also used. 

BPRS is an 18-item rating scale used to assess a range of psychotic and affective symptoms on the basis of both observation of the subject and subject’s own report. In some studies, a four-item BPRS positive symptoms subscale (BPRS+) is used [6], which considers suspiciousness, hallucinations, unusual thought content, and conceptual disorganization. BPRS and BPRS+ are used to assess treatment-emergent psychotic symptoms. Each symptom is rated on a scale from 0 to 6 (0: not present, 6: extreme).

When using the BPRS+, no symptoms or adverse events of psychosis were reported in patients administered intranasal esketamine [3].

The CADSS includes a 19-item scale used to evaluate patients’ own (subjective) answers and an 8-item scale used by a trained physician to assess patients’ responses during ketamine intake (objective). Worth noting is that the subjective items include three components: depersonalization, derealization, and amnesia [19]. With intranasal administration of esketamine, present-state dissociative symptoms, as measured by CADSS, began shortly after the start of esketamine treatment, peaked at 40 min, and generally resolved by 1.5 h. The magnitude of the symptoms attenuated with repeated administrations over time in the induction phase, with a relatively low magnitude reported in the optimization and maintenance phases. Thus, the intensity of the dissociative symptoms seems to be related to the treatment phase and/or the duration of drug exposure. 

Overall, 14 of 57 (25%) participants reported transient dissociative symptoms. [6].

Perceptual changes and/or dissociative symptoms, as measured by the CADSS, began shortly after the start of esketamine intranasal administration, peaked at approximately 30 to 40 min, and resolved by 2 h. Perceptual changes/dissociative symptoms attenuated in all dose groups with repeated dosing.

PWC-20 is a 20-item scale, shorter than the original 35-item PWC, used to assess withdrawal symptoms. In addition, it can be used to evaluate CNS symptoms and the effects of short-term interventions for the treatment of patients with suicidal behavior, since it allows the assessment of five specific domains, i.e., somatic, mood, cognitive, fatigue, and gastrointestinal outcomes linked to anxiety that can develop in patients during ketamine intake [22]. Evidence suggests that short- or long-term use of esketamine nasal spray is highly unlikely to be associated with withdrawal syndrome, as shown by the scores regarding stability, frequency, onset, and severity assessed by PWC-20 [23,24].

These questionnaires allow only data transparency but also comparisons between their results. 

### 2.3. Central Nervous System Symptomatology with Add-on Ketamine in TRBD Patients

Ketamine/esketamine intake is associated with wide-range adverse events, including cardiovascular (i.e., increase of blood pressure), ophthalmologic (i.e., blurry vision, diplopia), neurologic (i.e., dysgeusia, paresthesia, vertigo), psychiatric (i.e., somnolence, confusion, anxiety, sedation), and general (i.e., throat irritation, nasal discomfort, headache) effects [3]. Some of them can be present at the same time. Although the adverse effects of ketamine may be pleomorphic, this paper will focus on CNS safety.

We divided the symptoms of ketamine adverse effects in four major groups. In most of the reported studies, the majority of patients who were administered ketamine experienced non-significant dissociative symptoms [25] (Table 1).

### 2.4. Clinical Relevance

The most frequent CNS-related effects were dissociative symptoms. They most appeared shortly after dosing and seemed to be short in duration, completely resolving in ca. 2 h. Nevertheless, they were unpleasant for some patients, even causing withdrawal of consent [14], despite drug tolerance, with lower intensity of adverse events, developed with time [26] (Table 2). Some researchers showed that the presence of dissociative symptoms is a marker of antidepressant effectiveness [3]. The BPRS and BPRS+ scales indicated the absence of psychosis during the time of assessment [4,25].

### 2.5. Indications for Best Practice

According to our experience, it would be important to educate physicians about adverse effects that may appear more or less likely depending on individual patients’ status. This highlights the importance of monitoring patients before and after ketamine intake. Management in case of adverse events is rarely needed, but monitoring would allow to timely recognize and treat adverse events. Adverse events resolution mostly occurs spontaneously, and the severity of adverse events is dose-dependent, occurring at the moment of infusion and generally lasting for a short period of time (reaching a peak after 40 min from infusion, lasting up to ca. 2 h). If the adverse events do not resolve on their own, they could be treated with benzodiazepines such as lorazepam dosed ‘as needed’ (e.g., 1 mg, orally), which seems to completely resolve adverse events such as anxiety [13] (Table 3). It is worth to remind special populations who should not be automatically excluded from ketamine administration due to their comorbidities. The findings in a study by Zarate et al. states that ketamine, in the first 40 min after administration, has a swift and continuous anti-suicidal effect. However, this observation was not replicated when an intranasal esketamine formulation was administered. Another special population needing examination during ketamine administration is represented by TRBD patients, as reported in some studies [3,12,15]. Guidelines of the International College of Neuro-Psychopharmacology (CINP) for Bipolar disorder in adults (CINP-BD-2017) indicate ketamine as an option to treat bipolar depression in TRBD subjects [33].

## 3. Conclusions

CNS symptoms belong to the spectrum of adverse effects of ketamine use in TRBD patients that shall be adequately addressed during the treatment. Adverse events appear to be generally well tolerated, ephemeral, tending to return to baseline within 0.5–4 h after ketamine administration. The latest data on ketamine use in TRBD patients (including those related to the use of the recently approved esketamine nasal spray for patients with TRD–MDD) who experienced remission or response after esketamine treatment, show evidence that long-term treatment can be carried out using esketamine nasal spray, in addition to oral administration. The treatment appears to be clinically effective in preventing relapse in both short-term and the long-term interventions (maintenance treatment). Moreover it is necessary to establish guidelines for an effective and safe administration of ketamine to ‘special populations’ of patients who present common TRBD comorbidities, which often worsen the course of TRBD.

## Figures and Tables

**Table 1:** Symptomatology of CNS adverse effects of ketamine [12,15,17,26].

Group	Examples
Psychiatric	Dissociative symptoms (distortion of reality, feeling of bodily changes, acting as if dreaming, feeling of unreality or identity confusion),
Affective	Irritability, nightmares, agitation, anxiety, lethargy,
Cognitive	Global cognitive deterioration, sleepiness,
Neurologic	Transitory lack of motor coordination (duration <1 h), dizziness, headache, blurred vision

**Table 2:** Dissociative adverse events reported in trials for treatment-resistant depression (TRD)–major depressive disorder (MDD) and treatment-resistant bipolar depression (TRBD) [27,28,29,30,31,32].

Title				
‘Adverse events associated only with ketamine (≥10% of subjects) included dissociation, feeling strange, weird, or bizarre’	Diazgranados et al., 2010	Ketamine hydrochloride 0.5 mg/kg, 40 min infusion (single dose)	double-blind, randomized, placebo controlled, add-on	18 TRBD patients
‘Typical effects occurring at subanesthetic doses of ketamine were dissociation/perceptual disturbances, confusion (…); in no case did (…) dissociation persist beyond 60 min.’	Sos et al., 2013	Ketamine hydrochloride 0.54 mg/kg, 30 min (0.27 mg/kg for the first 10 min, followed by a maintenance infusion of 0.27 mg/kg within 20 min)	double-blind, randomized, placebo controlled	27 TRD-MDD patients, recurrent and single episodes
‘Eight of the 47 patients receiving ketamine (17%) had significant dissociative symptoms (i.e., feeling outside of one’s body or perceiving that time is moving more slowly or more quickly than normal) immediately after ketamine infusion; symptoms resolved by 2 h post-infusion. No severe psychotic symptoms (paranoia, hallucinations, delusions, or thought disorder) occurred in any patient.’ ‘There was no trend toward increasing dissociative or psychotomimetic effects over the course of the trial.’	Murrough et al., 2013	Ketamine hydrochloride 0.5 mg/kg, 40 min infusion	double-blind, randomized, with midazolam (active placebo)	24 TRD–MDD patients
‘Ketamine resulted in a mild increase in dissociative symptoms as measured by the Clinician-Administered Dissociative States Scale (increase from a mean of 0 before infusion to 8.60 ± 6.49 at the end of the infusion (t0 + 40 min); *p* = 0.0001), which returned to baseline by 120 min after infusion ends.’	Shiroma et al., 2014	Ketamine hydrochloride 0.5 mg/kg, 40 min infusion (up to 6 infusions, three times per week over a 12-day period)	Open-label	14 TRD-MDD patients
‘Intranasal ketamine was associated with small increases of measures of psychosis and dissociation. No relationship between ketamine-associated changes in dissociative or psychotomimetic symptoms and antidepressant response was found (*p* < 0.05 for CADSS and Brief Psychiatric Rating Scale).’	Lapidus et al., 2014	Ketamine hydrochloride 50 mg intranasal administration	Five intranasal applications of solution separated by 5 min each of five ketamine applications provided 10 mg of study drug.	20 TRD-MDD patients
‘Adverse events occurring during the infusion in 10% or more of the subjects receiving ketamine or placebo included feeling woozy or loopy, feeling lethargic or drowsy, cognitive impairment, fear or anxiety, nausea, dizziness, odd sensations, blurred vision, and headache. No adverse event was significantly different from placebo at 80 min or thereafter. Headaches, drowsiness, or sedation, early morning awakening, and difficulty falling asleep were reported in 10% of the sample in both the ketamine and the placebo phases. Dry mouth, dizziness or faintness, difficulty falling asleep, and flatulence were reported for ketamine only; irritability and muscle, bone, or joint pain were reported for placebo only. No significant changes occurred in electrocardiogram, respiratory, or laboratory values during the study.’	Zarate et al., 2012	Ketamine hydrochloride 0.5 mg/kg, 40 min. infusion	double-blind, randomized, placebo controlled, add-on	15 TRBD patients

**Table 3:** Indications for best practice.

Educate Your Colleagues about Ketamine Safety and Efficacy
Consider ketamine for TRBD patients (or TRBD ‘special populations’ and/or patients with suicidal thoughts)
Monitor TRBD patients, despite adverse events’ resolution is mostly spontaneous and uncomplicated
Treat adverse events only if necessary (f. ex. lorazepam 1 mg, orally)
